# Emergency endovascular repair of ruptured visceral artery aneurysms

**DOI:** 10.1186/1749-7922-2-17

**Published:** 2007-07-02

**Authors:** Umar Sadat, Nadim Noor, Tjun Tang, Kevin Varty

**Affiliations:** 1 Cambridge Vascular Unit, Addenbrooke's Hospital, Cambridge, UK

## Abstract

**Background:**

Visceral artery aneurysms although rare, have very high mortality if they rupture.

**Case presentation:**

An interesting case of a bleeding inferior pancreaticduodenal artery aneurysm is reported in a young patient who presented with hypovolemic shock while being treated in the hospital after undergoing total knee replacement. Endovascular embolization was successfully employed to treat this patient, with early hospital discharge.

**Conclusion:**

Prompt diagnosis and endovascular management of ruptured visceral aneuryms can decrease the associated mortality and morbidity.

## Background

Visceral artery aneurysms are an uncommon pathology, with a potential for rupture. Splenic artery aneurysms (SAA) are the most common (60%), followed by hepatic (20%), superior mesenteric (5.9%) and tripod celiac (4%) artery aneurysms. An interesting case of a bleeding inferior pancreaticduodenal artery aneurysm is presented and its management is also discussed.

## Case presentation

**Figure 1:**
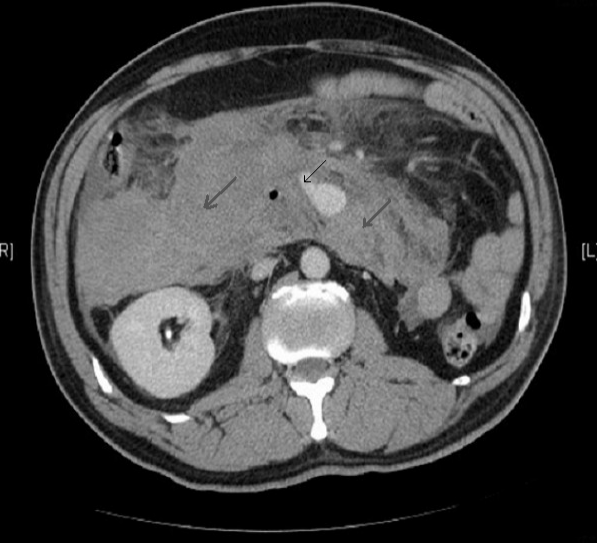
Contrast enhanced CT scan showing leak from the inferior pancreaticoduodenal arterial arcade with intramesenteric bleed.

Two days following a unicompartmental knee replacement, a 46-year old man developed acute abdominal pain radiating to the back with nausea. He rapidly deteriorated with tachycardia, hypotension and abdominal distension. Blood tests revealed low haemoglobin of 7 g/dl. After haemodynamic stabilization with IV fluids and blood, a CT scan was performed which showed a massive intramesenteric bleed and an aneurysm arising from the pancreaticodudoneal arterial arcade (Figure 1). Past medical history was unremarkable.

**Figure 2:**
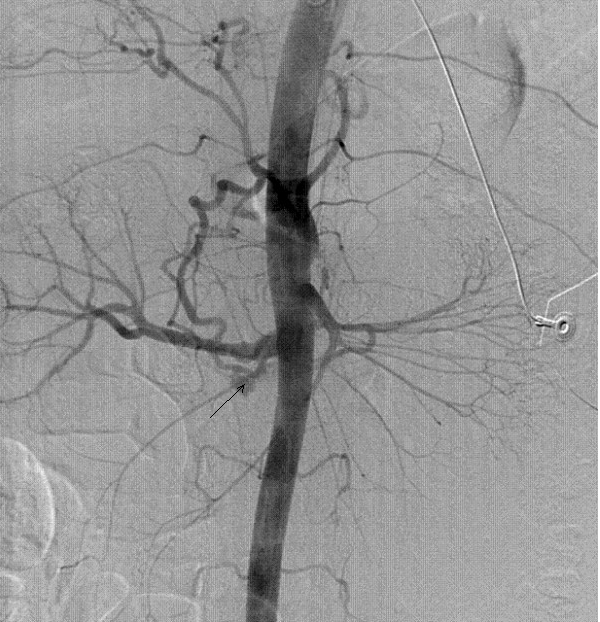
Digital subtraction angiogram showing contrast leak from the ruptured aneurysm.

**Figure 3:**
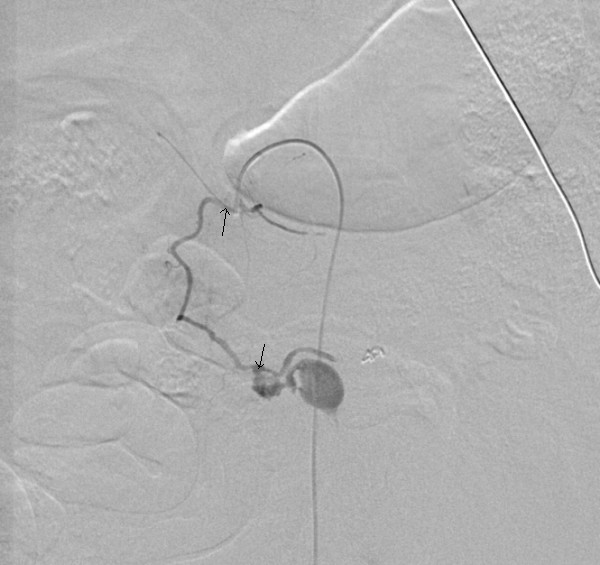
Coil embolization of feeding vessel through SMA.

**Figure 4:**
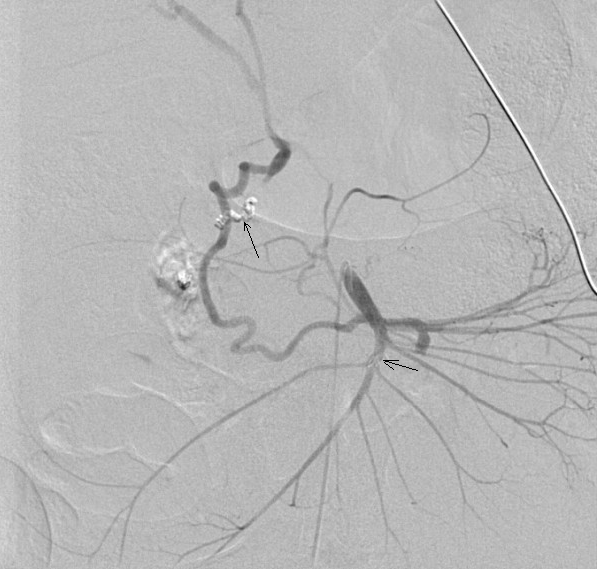
Angiogram showing successful coils embolization of the feeding vessel through celiac axis with no residual leak from the aneurysm.

Through a right-sided common femoral artery percutaneous cannulation, mesenteric arteriography was performed with the intention to perform coil embolisation. Images showed unusual distorted anatomy with a large and small gastroduodenal artery and an inferior pancreatico duodenal artery running parallel. There was extravasation of contrast from an aneurysm arising from the small duodenal vessels close to the superior mesenteric artery (SMA) (Figure 2). The feeding vessels were selectively embolised with coils from both SMA and celiac axis and stasis of flow was achieved (Figure 3, 4).

The patient stayed in critical care unit for 5 days because of cardio respiratory compromise resulting from massive intramesenteric bleed pre-procedure with haemoglobin reaching 4 g/dl, stabilized by blood transfusion and also increased intrabdominal pressure that gradually returned back to normal. He was discharged from the hospital on 7^th ^postoperative day.

## Conclusion

Aneurysms of the pancreaticoduodenal artery are extremely rare entities – only about 1–2% of all visceral aneurysms. Although they may be present in the absence of any risk factors, as happened in our case, however nearly 30% of these aneurysms evolve as complications of acute or chronic pancreatitis [1], of which 10% occur in patients with chronic pancreatitis [2]. The enzymes released from the pancreatic pseudo cyst frequently involve the splenic artery (45%) followed by the gastro-duodenal artery (17%) and then the pancreaticoduodenal artery (11%) [3]. Elastase can erode the artery wall leading to aneurysm formation [4]. When these aneurysms develop in the absence of pancreatitis, they are asymptomatic. Some are diagnosed as incidental findings on CT scans or arteriograms. If they present with acute retroperitoneal or intraperitoneal haemorrhage, as in this case, the reported mortality rates approach 20%.

These aneurysms can be managed surgically or by minimally invasive endovascular techniques with transcatheter embolization as done in this case or by a combination of both [5]. However, lately they have been successfully managed by thrombin injection [6,7] when tortuous anatomy makes embolization not feasible or using ethylene vinyl alcohol injection [8]. On extremely rare occasions they can thrombose on their own [9]. Surgical management is always challenging owing to the presence of multiple small pancreatic vessels that communicate with these aneurysms and may involve pancreatic resection. Minimally invasive coil embolization is therefore an attractive option and should be the first line of management, however it should be borne in mind that because in elective cases this approach has a 15% failure rate hence in a ruptured case this failure rate is likely to be higher.

## Conflict of interests

The author(s) declare that they have no competing interests.

## Authors' contributions

All the authors have been involved in literature search, writing and approval of final manuscript.
